# P-708. Clinical characteristics and outcomes among critically ill children aged 8 to 24 months with RSV lower respiratory tract infection by nirsevimab eligibility status – October 2023 – April 2025

**DOI:** 10.1093/ofid/ofaf695.920

**Published:** 2026-01-11

**Authors:** Laura D Zambrano, Margaret M Newhams, Regina Simeone, Amanda B Payne, Amber Orzel-Lockwood, Natasha B Halasa, Jemima Calixte, Katherine N Lindsey, Angela P Campbell, Adrienne G Randolph

**Affiliations:** Centers for Disease Control and Prevention, Atlanta, GA; Boston Children's Hospital, Boston, Massachusetts; Centers for Disease Control and Prevention, Atlanta, GA; CDC, Atlanta, Georgia; Boston Children's Hospital, Boston, Massachusetts; Vanderbilt University Medical Center, Nashville, TN; Boston Childrens Hospital, Boston, Massachusetts; Centers for Disease Control and Prevention, Atlanta, GA; Centers for Disease Control and Prevention, Atlanta, GA; Boston Children's Hospital, Harvard Medical School, Boston, Massachusetts

## Abstract

**Background:**

RSV is the leading cause of infant hospitalization, and some children with underlying conditions are at increased risk of severe RSV during their 2^nd^ RSV season. The Advisory Committee on Immunization Practices recommends nirsevimab for U.S. children in their 2^nd^ season with specified risk factors (Table 1). Criteria for 2^nd^ season eligibility vary by country. Our objectives were 1) to understand the proportion of nirsevimab-eligible children and 2) to describe underlying conditions and outcomes among those not eligible in a cohort of U.S. children with severe RSV lower respiratory tract infection (LRTI).

**Figure:**
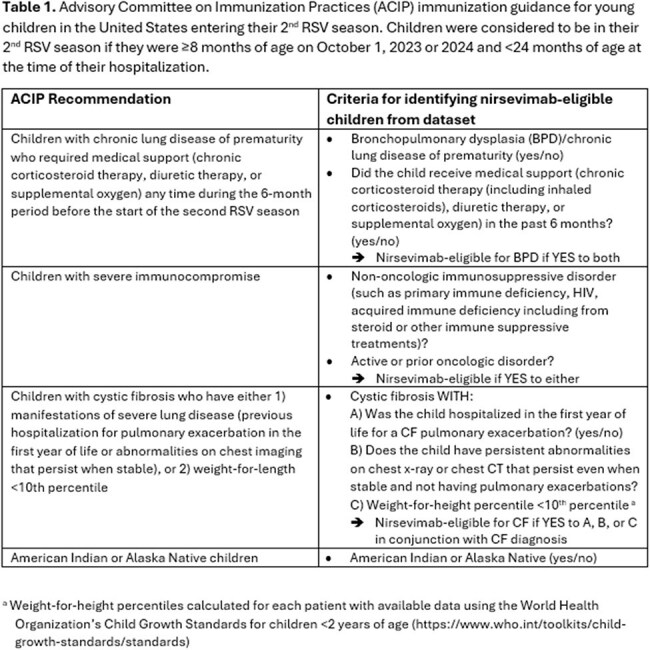

**Figure:**
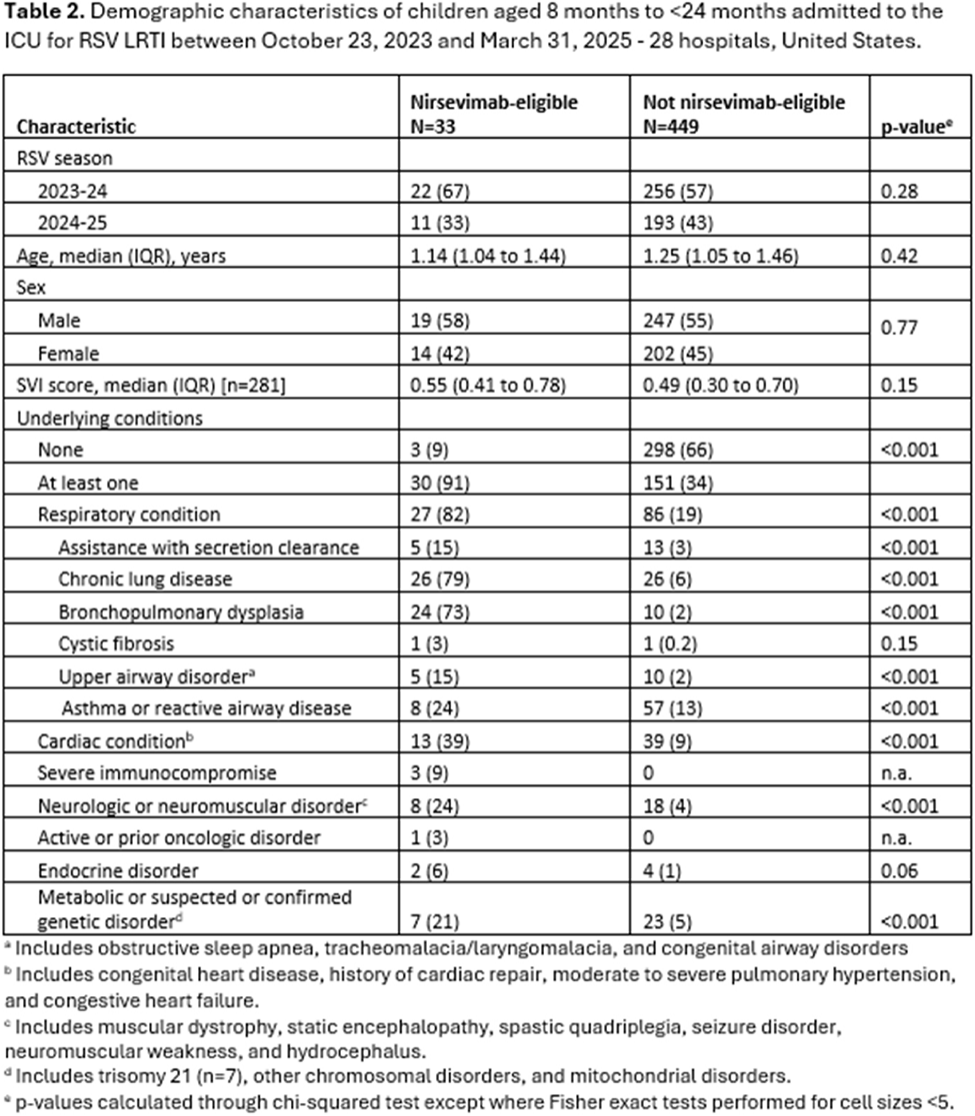

**Methods:**

We enrolled children aged 8−24 months hospitalized across 28 hospitals in 24 states with severe RSV LRTI during their 2^nd^ RSV season during October 30, 2023–April 12, 2024, and October 1, 2024–March 31, 2025. Analytic inclusion required 1) admission to the ICU for ≥24 hours and 2) high flow nasal cannula or noninvasive (continuous positive airway pressure [CPAP] or bilevel positive airway pressure [BiPAP]) or invasive ventilation. Patient characteristics and outcomes were compared by nirsevimab eligibility status using chi-square or exact tests.

**Figure:**
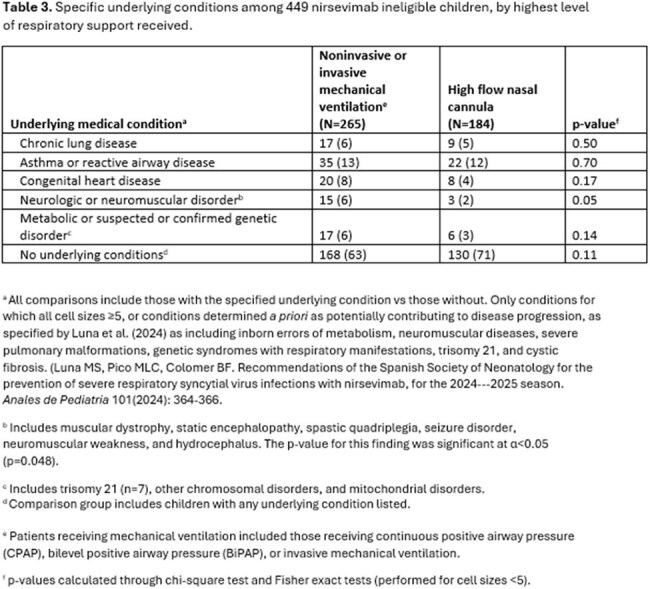

**Results:**

Among 482 children with severe RSV, 33 (6%) met 2^nd^ season nirsevimab eligibility: 5 (15%) were American Indian/Alaska Native, 24 (73%) had bronchopulmonary dysplasia (BPD) requiring ongoing medical support, one (3%) had cystic fibrosis with severe lung disease, and 3 (9%) had severe immunocompromise (Table 2). Many had other comorbidities, including 39% and 24% with cardiac and neurologic/neuromuscular disorders, respectively. Among 449 ineligible children, 151 (34%) had at least one underlying condition, and receipt of noninvasive or invasive ventilation was associated with neurologic or neuromuscular disease (p=0.048) (Table 3). Three children received nirsevimab in their 2^nd^ RSV season, including 2 children with BPD and one with chronic lung disease and congenital heart disease.

**Conclusion:**

Most children with ICU admission for severe RSV-LRTI during their 2^nd^ RSV season were ineligible for nirsevimab. Among those who were eligible, most qualified through having BPD. One-third of ineligible children with life-threatening illness had one or more respiratory, cardiac, and neuromuscular comorbidities.

**Disclosures:**

Natasha B. Halasa, MD, CSL-Seqirus: Advisor/Consultant|Merck: Grant/Research Support Adrienne G. Randolph, MD, MSc, Thermo Fisher, Inc.: Advisor/Consultant|UpToDate Inc.: Section editor, Pediatric Critical Care

